# Enzyme-catalyzed biodegradation of penicillin fermentation residues by β-lactamase OtLac from *Ochrobactrum tritici*

**DOI:** 10.1186/s12934-021-01606-2

**Published:** 2021-06-13

**Authors:** Peng Wang, Chen Shen, Qinqin Cong, Kaili Xu, Jialin Lu

**Affiliations:** 1 College of Chemical and Pharmaceutical Engineering, Hebei University of Science and Technology, Shijiazhuang, 050018 China; 2 State Key Laboratory Breeding Base-Hebei Province Key Laboratory of Molecular Chemistry for Drug, Hebei University of Science and Technology, Shijiazhuang, 050018 China; 3 Hebei Province Pharmaceutical Chemical Engineering Technology Research Center, Shijiazhuang, 050018 China

**Keywords:** β-Lactamase, Biodegradation, Antibiotic, Penicillin V, *Ochrobactrum tritici*

## Abstract

**Background:**

Biodegradation of antibiotics is a promising method for the large-scale removal of antibiotic residues in the environment. However, the enzyme that is involved in the biodegradation process is the key information to be revealed.

**Results:**

In this study, the beta-lactamase from *Ochrobactrum* *tritici* that mediates the biodegradation of penicillin V was identified and characterized. When searching the proteins of *Ochrobactrum* *tritici*, the β-lactamase (OtLac) was identified. OtLac consists of 347 amino acids, and predicted isoelectric point is 7.0. It is a class C β-lactamase according to BLAST analysis. The coding gene of OtLac was amplified from the genomic DNA of *Ochrobactrum* *tritici*. The OtLac was overexpressed in *E. coli* BL21 (DE3) and purified with Ni^2+^ column affinity chromatography. The biodegradation ability of penicillin V by OtLac was identified in an in vitro study and analyzed by HPLC. The optimal temperature for OtLac is 32 ℃ and the optimal pH is 7.0. Steady-state kinetics showed that OtLac was highly active against penicillin V with a Km value of 17.86 μM and a kcat value of 25.28 s^−1^ respectively.

**Conclusions:**

OtLac demonstrated biodegradation activity towards penicillin V potassium, indicating that OtLac is expected to degrade penicillin V in the future.

## Introduction

Antibiotic fermentation residue is the mycelium and culture medium remaining after the end of antibiotic fermentation production [1]. As it contains a large amount of unutilized nutrients such as glucose, corn pulp, peptone, beef extract, and mycelium protein, antibiotic residue is a biological resource with high nutritional value that is easy for organisms to use [1–3]. The traditional feed industry uses antibiotic fermentation waste as a feed additive and manufactures hyphae into animal feed additives to feed the animals. However, because these antibiotic bacterial residues contain antibiotics, untreated bacterial residues will cause these antibiotics to accumulate in animals or humans for a long period of time after entering the biological chain, which will lead to drug resistance and cause damage to the environment and ecological flora [4]. Therefore, antibiotic bacterial dregs are regarded as hazardous solid waste, and the use of environmentally friendly methods to process and recycle waste hyphae has become a difficult problem for the companies involved, and it is also an urgent problem for environmental workers.

Penicillin is one of the most widely used antibiotics and it is mainly produced by microbial fermentation [5]. The treatment of penicillin fermentation residues has become a major problem for pharmaceutical companies [6]. Traditional methods for penicillin fermentation residue degradation mainly include chemical methods [7], membrane separation [8], activated carbon adsorption [9] and photodegradation [10]. Various traditional bacterial residue treatment methods, including incineration, landfilling and anaerobic digestion [11, 12] have also been used. However, because these methods are prone to environmental pollution and inefficient, they have been gradually replaced by various advanced methods. Most recently, with the advancement of technology, many new physical and chemical methods have been developed for the degradation of penicillin antibiotics. For example, the use of photoelectric-Fenton oxidation (PEF) technology for the treatment of ampicillin [13]; the use of ionizing radiation to degrade penicillin [14]; and the use of UV/TiO_2_ photocatalytic degradation of amoxicillin can achieve amoxicillin degradation. Penicillin can be degraded by nano Fe/Ni (B-Fe/Ni) supported by functional bentonite [15], Zinc-Loaded materials on a goethite surface [16], CoFe_2_O_4_@CuS magnetic nanocomposite and so on [17]. However, these methods are costly, the processing conditions are harsh, and it is difficult to scale up.

Microbiological degradation has become an effective way to address antibiotic contamination due to its advantages, such as high efficiency, low cost and simple operation [18]. This biodegradation method is mainly completed by microbial flora or a certain kind of bacteria in the activated sludge. In recent years, various strains isolated from the environment have been chosen as candidates for the degradation of antibiotic residues [19]. For example, microorganisms isolated from wetland plant root sediments was designed for the treatment of livestock wastewater containing traces of fluoroquinolones and cephalosporins [20]. According to reports, bacteria that can be used for biodegradation of penicillin antibiotics include *Pseudomonas *[21], Shewanella [22], Bacillus [23], Klebsiella [24] and *Paracoccus *[25]. However, currently, strain screening and cell-level degradation remain among the studies of the biodegradation of penicillin, and there are few reports from the perspective of the enzymatic and molecular levels, preventing researchers and engineers from developing more efficient and environmentally friendly enzymatic degradation processes.

Enzymes that have been reported to be involved in the biodegradation of penicillin included β-lactamase and penicillin acylase [26]. However, the research on β-lactamase has mainly aimed at mediating the occurrence of drug-resistant bacteria [27, 28] and the research on penicillin acylase has mainly aimed at the synthesis of synthetic penicillin antibiotics [29, 30]. As the demand for penicillin antibiotic slag detoxification increases, it is necessary to find more β-lactamases for the biodegradation of penicillin antibiotics. Since the enzyme catalysis process will not cause the spread of resistance genes or the occurrence of drug-resistant strains, the application of β-lactamase in the field of harmless antibiotic treatment will make this kind of enzyme friendly for applications in environmental governance and sustainable development.

One of the commonly used slow-onset antibiotics is penicillin V potassium (PVK) which was used for the treatment of various bacterial infections [31]. Compared to natural penicillin, PVK is more acid resistant to acid, so PVK can be taken orally and is not hydrolyzed by gastric acid. In addition, because PVK has good resistance to hydrolysis, the stability of treating bacterial infections is improved [32]. However, due to the large and widespread use of PVK, high concentrations of PVK have been found in soil, wastewater, and even livestock and poultry manure, which poses a serious threat to the ecological environment and human health, and may cause the appearance of related resistant strains. Therefore, researchers are looking for ways to find the degradation of PVK residues in the environment [23].

In our previous studies, a highly efficient degradation strain for penicillin V potassium was isolated from the activated sludge by directional breeding [33]. This strain was identified as *Ochrobactrum* *tritici*. In the present study, the β-lactamase candidated in the biodegradation of penicillin V potassium was identified from the genome of *Ochrobactrum* *tritici*. The β-lactamase was heterologously expressed and purified. The optimal temperature and pH were studied for enzymatic catalysis. This work provides a novel candidate for penicillin degradation by enzymes.

## Materials and methods

### Materials

Penicillin V potassium was penicillin G were purchased from Mecklin. IPTG, NaCl and imidazole were purchased from Mecklin (Shanghai, China). Yeast extract and peptone were purchased from Sigma-Aldrich (St. Louis, MI, USA). All other chemicals, reagents, and solvents such as Na_2_HPO_4_, NaH_2_PO_4_ and acetonitrile, etc were all purchased from Mecklin (Shanghai, China) and have the highest purity.

### Identification of the β-lactamase from *Ochrobactrum tritici*

Firstly, the NCBI database was searched to find out β-lactamase coding genes form *Ochrobactrum* *tritici*. Then, dependent on the searched sequences, the primers were designed for amplifying β-lactamase coding gene in *Ochrobactrum* *tritici* X-2 from our lab. And the coding gene was amplified from the genomic of *Ochrobactrum* *tritici* X-2 and sequenced to obtained its nucleotide sequence.

### Plasmid construction

The genomic DNA of *Ochrobactrum* *tritici* was extracted using Rapid Bacterial Genomic DNA Isolation Kit (Sangon Biotech China Co., Ltd. Shanghai) The β-lactamase gene was amplified from genomic DNA of *Ochrobactrum* *tritici* using PCR with the primers F (ggaattccatatgAGAAAATCTACGACATTTTTG) and R (cggaattcTTATTGTTTCTTGTCGAGCGCC). Then the PCR product was cloned into pET28a under the restriction sites of *Nde*I and *Eco*RI to obtain pET28a-OtLac to express of OtLac with the 6-His tag at the N-terminus.

### Expression and purification of β-lactamase

To express the β-lactamase OtLac, *E. coli* BL21 (DE3) cells were used. *E. coli* BL21 (DE3) cells were transformed with the plasmid OtLac and inoculated in LB medium containing 10 μg/mL kanamycin. The *E. coli* BL21 (DE3) cells were cultured at 37 ℃ and 200 rpm until the OD600 reached to 0.4–0.6 IPTG (0.1 mM final concentration) was added to the culture for OtLac expression *E. coli* cells were grown for 12 h at 20 ℃ with shaking at 200 rpm. After the induction, the bacteria were collected by centrifugation at 5000 rpm, and the *E. coli* cells were crushed by ultrasonic disruption. The crushed solution was centrifuged at 12000 rpm for 10 min. The supernatant was taken as the crude protein extract for OtLac purification.

OtLac fused with a His tag was purified by using Ni^2+^-NTA column purification. First, a crude histidine-tagged recombinant OtLac protein extract was loaded onto a nickel ion exchange column. Then, two column volumes were eluted with the eluent A (containing 50 mM NaH_2_PO_4_ and 300 mM NaCl, pH 8), and the eluent B (containing 50 mM NaH_2_PO_4_, 300 mM NaCl and 10 mM imidazole, pH 8). These two column volumes were used for elution of the non-specific binding proteins. The target protein, His-tag fused OtLac, was then eluted with two column volumes (cv) of the eluent C (containing 50 mM NaH_2_PO_4_, 300 mM NaCl and 50 mM imidazole, pH 8). After purification, the eluent C was changed with 50 mM pH 7.4 sodium phosphate buffer by using a 30 kDa cut-off molecular weight cut-off (MWCO) ultrafiltration tube.

### In vitro biocatalysis assay

Using a reconstituted system using OtLac as the biocatalyst, the substrate conversion in vitro was conducted in vitro. The in vitro catalytic system contained β lactamase (0.5 μM) in 0.1 M pH 7.4 sodium phosphate buffer. The substrate penicillin V or penicillin G was dissolved in sodium phosphate buffer and added to the in vitro catalytic system so that the final concentration of antibiotic reached 250 μM. The total reaction volume was 250 μL, and the reaction temperature was 28 ℃. After a 30 min incubation, the reaction was stopped by adding ethyl acetate (250 μL). Extraction was performed twice with ethyl acetate and then the organic extracts were combined, dried and analyzed by HPLC.

### Effect of temperature and pH on biocatalytic activity of β-lactamase

In this study, the optimal temperature for the biotransformation of penicillin V or penicillin G by purified OtLac was investigated in the temperature range of 20 to 37 ℃ at pH 7.0. Additionally, the influence of pH on whole cell biotransformation was studied in the pH range of 6.0–8.5 under the temperature of 28 ℃. The incubation time was 30 min.

### Steady-state kinetics assays

The kinetic parameters were estimated with a substrate (penicillin G or penicillin A) concentration range from 0 to 500 μM using 0.5 μM of OtLac. The in vitro biocatalysis was conducted in 0.1 M sodium phosphate buffer at pH 7.4. The reactions were started by adding certain concentration of OtLac and quenched after 5 sec by adding two reaction volumes of methanol, and the concentration of the product was determined by HPLC. The Km and kcat values were calculated by plotting the substrate conversion velocities against the substrate concentrations using Michaelis-Menten kinetics and utilizing GraphPad Prism 5 software.

### Batch treatment of industrial penicillin fermentation residues by β-lactamase OtLac from *Ochrobactrum tritici*

First, the penicillin bacteria residue was washed three times with water, and ultrasonic treatment was performed during the washing process to fully release the penicillin and other substances in the penicillin bacteria residue. The pH value of the washing liquid was adjusted to neutral (pH = 6.8–7.2), and β-lactamase OtLac was added to the washing liquid. The final concentration of the enzyme was 0.25 mg/mL. Samples were taken at different time intervals (0 h, 2 h, 6 h, 12 h, 18 h, 24 h, 36 h and 48 h), and the residual amount of penicillin in the washing liquid was determined by HPLC.

### HPLC analysis

The degradation products of penicillin V potassium were analyzed using an Agilent 1260 HPLC instrument. The degradation products and substrate PVK were separated on a reversed-phase C18 column at a flow rate of 1 mL/min. Mobile phase A was 0.5 M potassium dihydrogen phosphate (pH was adjusted to 3.5 using phosphate), mobile phase B was deionized water and mobile phase C was methanol. The ratio of mobile phases A, B and C was set as 1:4:5. The column oven temperature was 30 °C, and the UV absorption was 225 nm.

## Results and discussion

### Β-Lactamase from ***Ochrobactrum tritici***

**Fig. 1:**
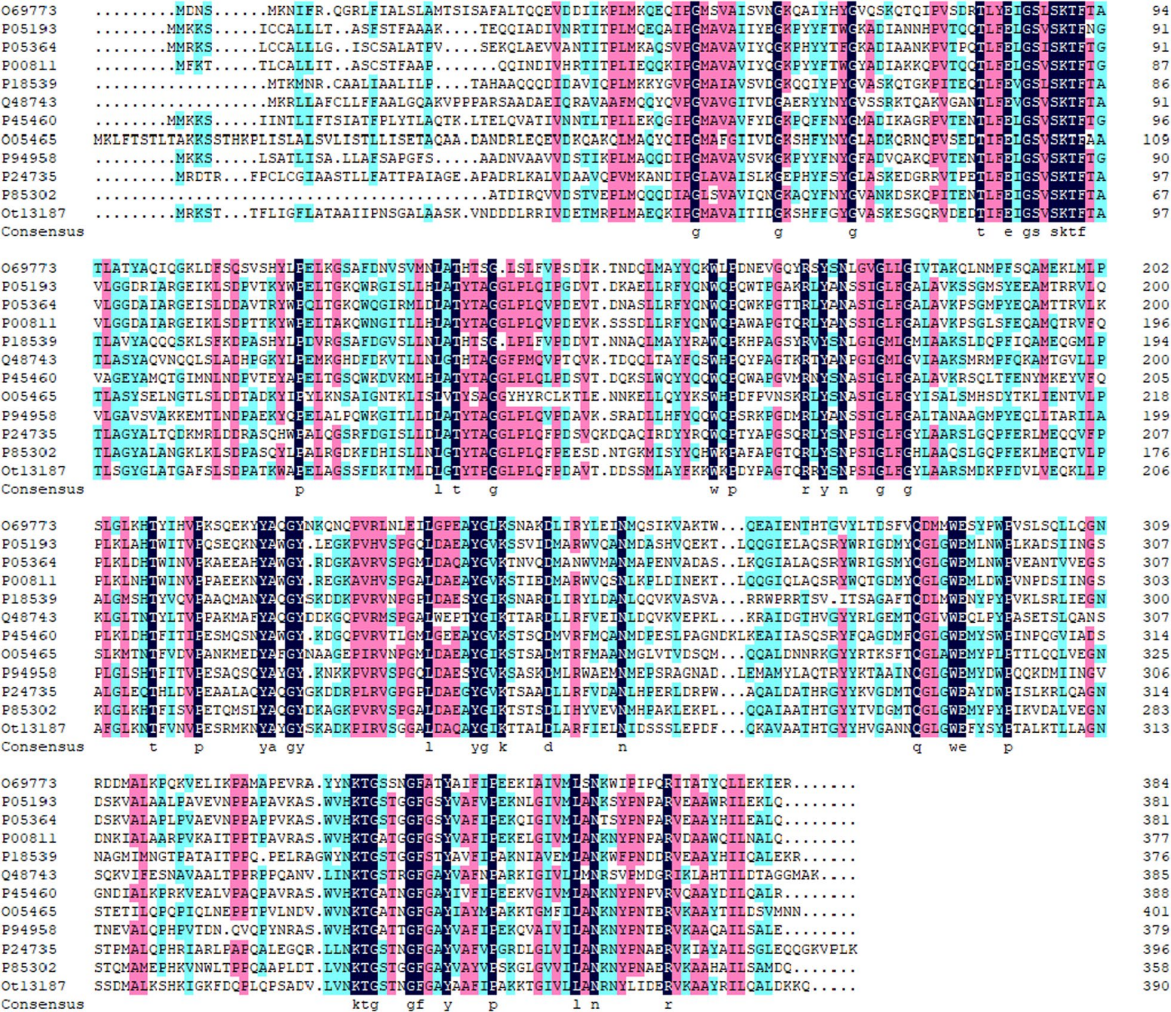
Sequence alignment of OtLac and various β-lactamase. Amino acid sequence alignment was performed using ClustalW. (The selected sequences: P05193: *Citrobacter freundii*; P00811: *Escherichia* *coli* (strain K12); P05364: *Enterobacter cloacae*; P45460: *Yersinia enterocolitica*; P94958: *Morganella morganii*; P24735: *Pseudomonas aeruginosa*; P85302: *Pseudomonas fluorescens*; O05465: *Psychrobacter immobilis*; Q48743: *Lysobacter lactamgenus*; O69773: *Providencia stuartii*; P18539: *Serratia marcescens*)

**Fig. 2:**
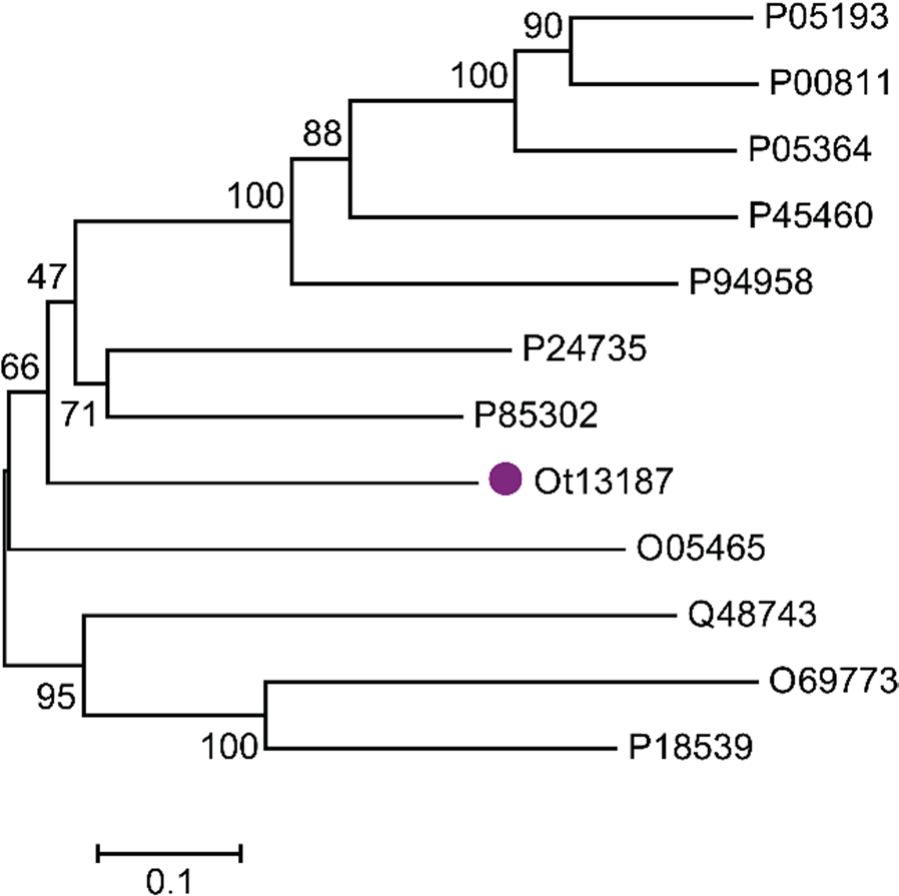
Phylogenetic tree of OtLac (Ot13187) from *Ochrobactrum tritici* and other functionalized β-lactamases from the Swiss-Prot database. Amino acid sequence alignment was performed using ClustalW. The tree was constructed using a neighbor-joining algorithm and 1000 repeated guided analyses. Guide values are displayed on the branch nodes. The scale bar represents 0.2 amino acid substitutions per amino acid. The OtLac (Ot13187) from *Ochrobactrum* *tritici* is indicated with the closed circle (●)

According to the sequence alignment analysis, the sequence of OtLac showed strong homology with class C group β-lactamase, thus, OtLac was predicted to be a class C group β-lactamase. The amino acids aligned with the transpeptidase superfamily. Most members of the class C β-lactamase family are ampicillin hydroxylases. From pairwise alignments, OtLac also had a high amino acid sequence identity with the sequences of accession number P85302, P24735, P94958, O05465 and P45460, showing 55.90%, 51.70%, 46.93%, 46.91% and 46.32% identity, respectively. This high identity suggests that OtLac is a member of the β-lactamase family and hydrolyses the lactam ring. The sequence alignment between OtLac and various identified β-lactamase from the Swiss-Prot database is shown in Fig. 1, and the phylogenetic analysis is shown in Fig. 2.

### Cloning and recombinant expression of β-lactamase

**Fig. 3:**
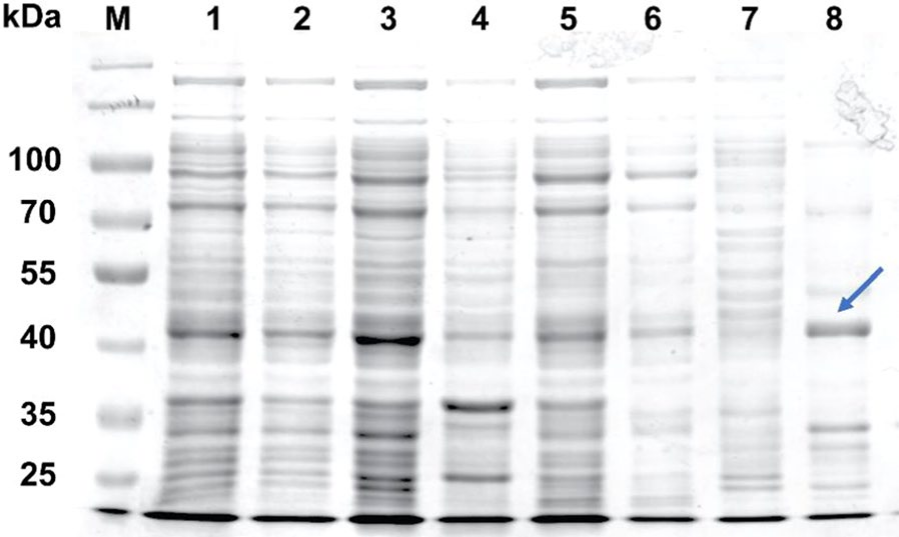
Expression and purification of OtLac (SDS-PAGE of expression and purification of OtLac, protein samples were taken at different stages of expression and purification. Lane M, prestained protein marker. Lane 1, cell lysate before induction with IPTG. Lane 2, cell lysate after 24 h of expression. Lane 3, cell free supernatant after sonication. Lane 4, precipitation of cells. Lane 5, Proteins that do not bind to Ni^2+^ gel. Lane 6, 2 mM imidazole eluent. Lane 7 10 mM imidazole eluent. Lane 8, purified OtLac) with 50 mM imidazole eluent

The coding gene of OtLac was amplified from the genomic DNA of *Ochrobactrum* *tritici* using PCR. The PCR product was approximately 1.2 kb. Then, the PCR product of OtLac was cloned into the pET28a vector. *E. coli* BL21 (DE3) cells were used for the efficient expression of OtLac. IPTG was added for inducing the OtLac expression. The expression of OtLac was analysed by SDS-PAGE which is shown in Fig. 3C.

### Purification of β-lactamase

In this study, nickel affinity chromatography was used to purify the histidine-tagged fused OtLac. The OtLac gene was cloned into the *Nde*I and *Eco*RI cloning sites in the vector pET28a (+) to generate a recombinant protein with an N-terminal His6 tag. *E. coli* BL21 (DE3) was cultured at 20 ℃ for protein expression, yielding a recombinant protein of up to 14% of the total soluble protein with a molecular weight of 43 kDa. A metal chromatography column was used to elute the protein sample, and a 50 mM imidazole eluate was collected to obtain a pure enzyme, as shown in Fig. 3C. The protein was concentrated using an ultrafiltration membrane. By Bradford analysis, 140 mg of pure OtLac can be obtained from 500 mL of culture and the purity of the enzyme was determined to be 92.81%.

### In vitro biotransformation of penicillin V and penicillin G by OtLac

**Fig. 4:**
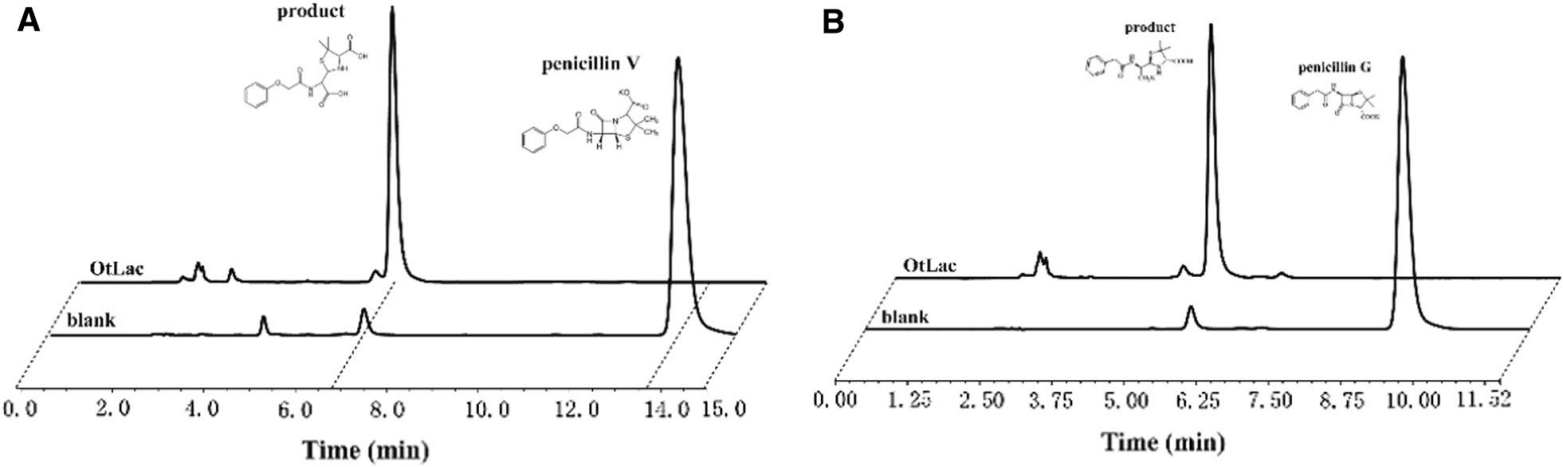
In vitro biodegradation of penicillin V and penicillin G by OtLac. **A** HPLC analysis of penicillin V degradation by OtLac, the retention time of penicillin V and its product was 13.71 min and 6.77 min respectively. **B** HPLC analysis of penicillin G degradation by OtLac, the retention time of penicillin G and its product was 9.23 min and 5.89 min respectively

In order to identify the biocatalytic ability and efficiency of penicillin V and penicillin G by OtLac, an in vitro biocatalytic study was conducted. A reaction mixture containing the β-lactamase was used for the in vitro bioconversion of penicillin V and penicillin G, while a reaction mixture without β-lactamase was used as a negative control. The biocatalytic properties of penicillin V or penicillin G were determined by HPLC analysis. A diode array detector (DAD) was used for substance detection. As shown in Fig. 4, only when the reaction system contained OtLac can the biotransformation products can be detected during the catalytic reaction, which indicates that OtLac has bioconversion penicillin G and penicillin V bioconversion activity. The penicillin V product had a retention time of 6.77 min, and we proposed that this was the corresponding lactam ring cleavage product. The penicillin G product retention time is 5.89 min, and we proposed that this was the corresponding lactam ring cleavage product.

### Influence of temperature and pH on biocatalytic activity of the β-lactamase from *Ochrobactrum tritici*

**Fig. 5:**
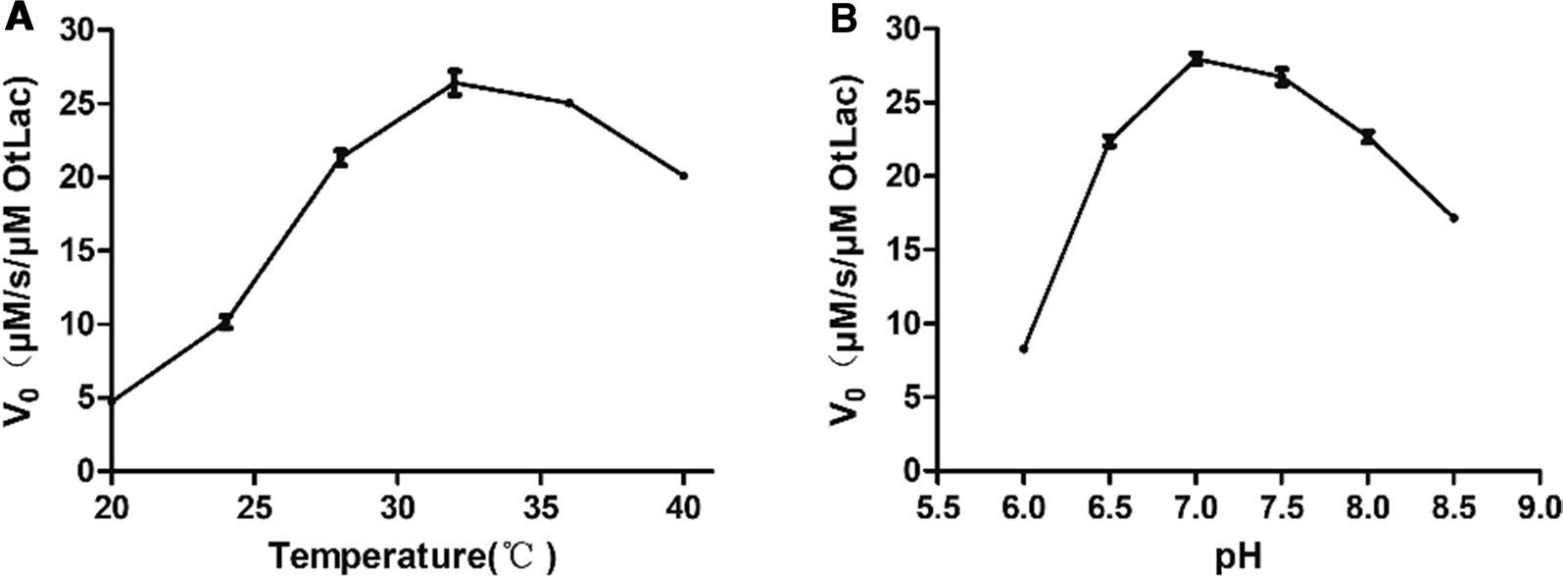
Effects of temperature and pH on the biocatalytic activity of OtLac. The incubation time was 30 min. And the pH test was conducted under the temperature of 28 ℃

The optimal temperature for OtLac activity was investigated over the temperature range of 20–40 °C (Fig. 5A). The results indicated that the optimal temperature of OtLac was 32 ℃. Thermostability data showed that OtLac was thermolabile with an approximate half-life of 30 min at 40 °C. In order to study the optimal pH for OtLac, various pH values ranging from 6.0 to 9.0 were tested. The preferred pH of OtLac was over the range from 7.0 to 7.5.

### Kinetic assays of OtLac towards penicillin V and penicillin G

The kinetic constant of the enzymatic reaction of purified OtLac to penicillin V was studied. The tested concentrations of substrate PVK ranged from 10 to 300 μM, and the kcat, and Km values were 25.82 s^−1^, and 17.86 μM, respectively. The kinetic constant of the enzymatic reaction of purified OtLac to penicillin G was also studied. The substrate concentration was 10–500 μM, giving kcat, and Km values of 22.28 s^−1^, and 32.75 mM, respectively. These results suggested that OtLac was more efficient for penicillin V biodegradation.

β-Lactamases of class C are widely distributed on the chromosomes of many Gram-negative species. They can catalyze wide range of substrates like penicillin, Cephalothin, Meropenem, et al. In this study, it was the first report for this class of β lactamase for catalyze penicillin V. The comparison of OtLac with other class C β-lactamase for different substrates was shown in Table 1.

**Table 1:** comparison of OtLac with other class C β-lactamases

Enzyme	Substrate	Km (μM)	kcat	Reference
OtLac	Penicillin V	17.86	25.28	This study
ADC-1	Ampicillin	32	4.8	[34]
	Benzylpenicillin	5.1	10.26	
	Oxacillin	12.32	0.046	
	Piperacillin	6.4	3.1	
ACT-3 AmpC	Penicillin G	26		[35]
CMY-10	Benzylpenicillin	20.5	3.06	[36]
YOC-1	Benzylpenicillin	934.2	178,900	[37]
	Ampicillin	109.4	5533.33	
LHK-5	Benzylpenicillin	356		[38]

### Treatment of an industrial penicillin fermentation residue by OtLac

**Fig. 6:**
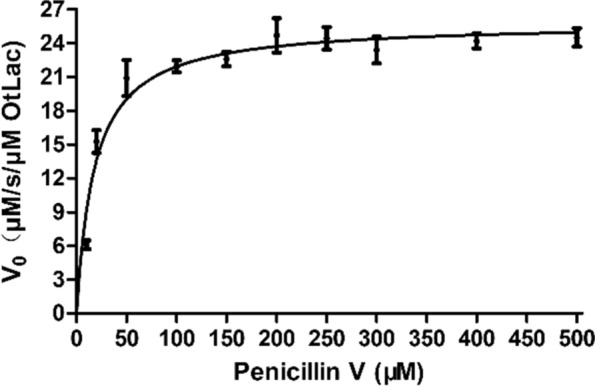
Determination of the Km value for penicillin V bio-catalyzed by OtLac. The relationship between the maximum reaction rate and the substrate concentration is plotted. GraphPad Prism 5 software was used to fit the average of three independent measurements

**Fig. 7:**
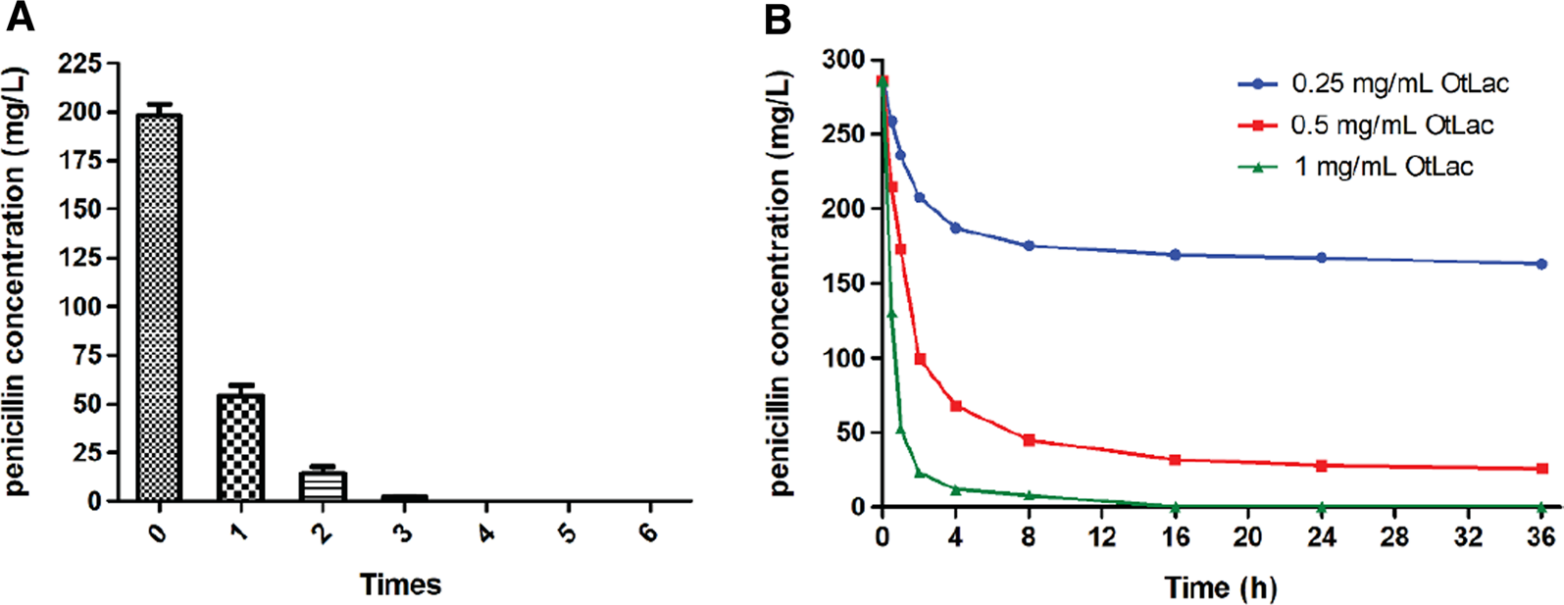
Biodegradation of industrial penicillin G fermentation residues by OtLac.** A** The relationship between penicillin concentration and extract times with ultrasonic.** B** Time course of industrial penicillin fermentation residue biodegradation by varies concentration of OtLac (0.25 mg/mL, 0.5 mg/mL, 1 mg/mL)

OtLac was used to treat the industrial penicillin fermentation residues. First, we investigated the optimal number of times for penicillin extraction from the residue by ultrasonic extraction. Ultrasonic extraction was repeated six times, and after three extractions, penicillin could no longer be detected in the extract water, as shown in Fig. 6A. Thus, the optimal number of extraction times was three. Next, the industrial penicillin fermentation-extracted water was treated with OtLac. Various concentrations (0.25 mg mL^−1^, 0.5 mg mL^−1^ and 1 mg mL^−1^) of OtLac were used for penicillin biodegradation. As shown in Fig. 6B, in the first 1 h, the biodegradation rate was the same among the tested enzyme concentrations, which were 200 mg h/mg^−1^, 226 mg h^−1^ mg^−1^ and 233 mg h^−1^ mg^−1^, respectively. However, an hour later, the biodegradation rate was slightly decreased and after two h, the biodegradation was significantly decreased. These phenomena indicated that the enzymatic activity could be maintained in the first hour but was severely inactivated after 2 h. When the concentration of OtLac was 1 mg mL^−1^, penicillin was easily degraded. Our study was conducted with free enzymes, which are easily influenced by factors in the external environment, such as pH, metal irons and other toxins. This problem can be solved by immobilization, degradation-separation coupling processed and even enzyme evolution methods (Fig. 7).

## Conclusion

In this study, the β-lactamase OtLac was identified and characterized from *Ochrobactrum tritici* was shown to possess biodegradation ability for both penicillin G and penicillin V. OtLac is a class C β-lactamase as analysed by sequence alignment. The biocatalytic efficiency for PG and PV of OtLac was much higher than that reported for other class C β-lactamases, making it a potential candidate for the enzymatic biodegradation of β-lactam antibiotic degradation in the environment.

## Data Availability

Not applicable.
